# Pathogenicity of highly pathogenic avian influenza H5N8 subtype for herring gulls (*Larus argentatus*): impact of homo- and heterosubtypic immunity on the outcome of infection

**DOI:** 10.1186/s13567-022-01125-x

**Published:** 2022-12-14

**Authors:** Karolina Tarasiuk, Anna Kycko, Małgorzata Knitter, Edyta Świętoń, Krzysztof Wyrostek, Katarzyna Domańska-Blicharz, Łukasz Bocian, Włodzimierz Meissner, Krzysztof Śmietanka

**Affiliations:** 1 Department of Poultry Diseases, National Veterinary Research Institute, Al. Partyzantów 57, 24-100 Puławy, Poland; 2 Department of Pathology, National Veterinary Research Institute, Al. Partyzantów 57, 24-100 Puławy, Poland; 3 Ornithology Unit, Department of Vertebrate Ecology & Zoology, Faculty of Biology, University of Gdansk, Wita Stwosza 59, 80-308 Gdańsk, Poland; 4 Department of Epidemiology and Risk Assessment, National Veterinary Research Institute, Al. Partyzantów 57, 24-100 Puławy, Poland

**Keywords:** Avian influenza, pathogenicity, herring gulls, homosubtypic immunity, heterosubtypic immunity

## Abstract

To improve understanding of the pathobiology of highly pathogenic avian influenza virus (HPAIV) infections in wild birds, pathogenicity and transmissibility of HPAIV H5N8 subtype clade 2.3.4.4b was evaluated in ~ 8-week-old herring gulls (*Larus argentatus*) divided into 3 groups: naïve birds (group A), birds previously exposed to low pathogenic avian influenza virus (LPAIV) H5N1 (group B) and LPAIV H13N6 (group C). The HPAIV H5N8 virus was highly virulent for naïve gulls, that showed early morbidity, high mortality, a broad spectrum of clinical signs, including violent neurological disorders, systemic distribution of the virus in organs accompanied by high level of shedding and transmission to contact birds. Pre-exposure to homologous and heterologous LPAIV subtypes conferred only partial protection: we observed increased survival rate (statistically significant only in group B), nervous signs, pantropic distribution of virus in organs, shedding (significantly reduced in gulls of group C in the early phase of disease and asymptomatic shedding in the late phase), transmission to contact gulls (more pronounced in group B) and near-complete seroconversion in survivors. Histopathological and immunohistochemical results indicate virus tropism for the neural, respiratory and myocardial tissues. In conclusion, we demonstrate that HPAIV H5N8 clade 2.3.4.4b is highly virulent and lethal for fully susceptible herring gulls and that pre-exposure to homo- and heterosubtypic LPAIV only partially modulates the disease outcome.

**Supplementary Information:**

The online version contains supplementary material available at 10.1186/s13567-022-01125-x.

## Introduction

Wild aquatic birds of the *Anseriformes* order (e.g. ducks, geese and swans), next to the *Charadriiformes* order (e.g. gulls, terns, shorebirds and auks), are the natural reservoir of avian influenza viruses (AIV), particularly low pathogenic avian influenza viruses (LPAIV) [1]. All known avian hemagglutinin (H) and neuraminidase (N) subtypes (i.e. H1–H16 and N1–N9) have been detected in both bird orders but with different incidence and distribution across species, mostly due the high degree of species-specificity that often occurs as a result of the host and virus co-evolution [2, 3]. The LPAIV of H5 and H7 subtypes can sometimes mutate to highly pathogenic form (known as highly pathogenic avian influenza—HPAI) and the worldwide losses notified in recurrent epizootics caused by HPAI viruses (HPAIV) belonging to so called “H5 Gs/Gd lineage” are increasing, both in domestic and wild populations [4]. The contribution of wild birds to HPAIV dissemination, disputable before 2014, has been a matter of consensus after repeated (and rapid) spread of the virus across Eurasia, Africa and North America in 2014–2022 and its abundant detection in wildlife populations. The role of wild birds in HPAI epidemiology is complex: they can serve as long-distance spreaders, maintenance reservoirs or “dead-end” hosts [5]. Many species of synanthropic birds are defined as “bridge” hosts contributing to the transmission of the virus from a wild reservoir population to poultry [6]. From the surveillance perspective, it is of particular importance to know whether wild birds are asymptomatic carriers or if they succumb to infection; in the latter case, enhanced passive surveillance targeted at defined species of birds is an important element of early virus detection. However, the binary division of birds into “asymptomatic carriers” and “sensitive sentinels” is somewhat simplified since it does not take account of complex host–pathogen interaction, e.g. age resistance, presence of co-infections or existence of partial (or complete) immunity acquired as a result of previous infection with antigenically similar virus.

Gulls and other representatives of *Laridae* family are important hosts for both LPAIV and HPAIV. For example, although virtually all H and N subtypes of LPAIV have been detected in gulls, H13 and H16 subtypes are particularly “gull-adapted” [7]. Experimental studies showed high morbidity and mortality following infection with early clades of HPAIV H5N1 subtype in herring gulls *Larus argentatus* [8], laughing gulls *Larus atricilla* [9] and black-headed gulls *Chroicocephalus ridibundus* [10]. Gulls have always been found among positive wild birds during the consecutive HPAI epizootics in Europe in the past years [11, 12]. That relates mostly to the HPAIV H5 variants of “Gs/Gd” lineage, classified to genetic clade 2.3.4.4b, that have been particularly prone to form combinations with different neuraminidase subtypes, most often with N1, N2, N5, N6 and N8, and therefore frequently referred to as “H5Nx” viruses [13]. For example, between the 2016/17–2020/2021 HPAI seasons, the following species of gulls have been affected by the HPAIV H5Nx clade 2.3.4.4.b: herring gull, black-headed gull, lesser black-backed gull *Larus fuscus*, great black-headed gull *Larus marinus*, common gull *Larus canus*, Armenian gull *Larus armenicus* [3]*.* In the most recent HPAI season in Europe (2021/2022), the list of affected species expanded and the H5 virus was also detected in yellow-legged gull *Larus michahellis*, Caspian gull *Larus cachinnans*, grey-headed gull *Chroicocephalus cirrocephalus*, western gull *Larus occidentalis*, glaucous gull *Larus hyperboreus* [14]. The virus has been detected mostly in dead birds but there are sporadic reports of HPAIV H5N1 detections in apparently healthy gulls [15, 16]. Interestingly, during the HPAI 2016/2017 season in Poland, an H5N8 HPAIV was detected in an apparently healthy herring gull that was found dead exactly 1-day later, thus suggesting that it had been in the incubation period at the time of sampling (authors’ unpublished data).

Herring gull is widespread species, which inhabits vast area from Iceland, the British Isles and northern France through northwest Europe to northwest Russia [17]. Northern populations are migratory and winter mainly in maritime northwest Europe, while populations in the south-western part of the breeding area are nomadic or completely non-migratory [18]. This species has a highly opportunistic diet and forages on a wide range of marine and terrestrial habitats as well as exploiting anthropogenic food sources from landfills, sewage outfalls, household wastes in urban areas and fishing waste [19–21]. Recently herring gulls make substantial use of anthropogenic resources and has increased in numbers in urban areas and is often observed feeding on food discarded by humans [22, 23].

The aim of the study was to investigate pathogenicity and transmissibility of HPAIV H5N8 (clade 2.3.4.4b) in herring gulls and to study the impact of preexisting immunity induced by homo- or heterosubtypic LPAIV infection (H5N1 and H13N6) on the severity of disease caused by the highly pathogenic virus.

## Materials and methods

### Viruses

The viruses used in the study originated from the repository of NVRI, Pulawy, Poland:

a) A/herring gull/Poland/MB082B/2016 (H5N8) HPAIV was isolated from a dead herring gull during the HPAI epizootic in Poland in the 2016/2017 season and belonged to Gs/Gd lineage, clade 2.3.4.4b (EPI_ISL_15430045) [24]

b) A/mallard/Poland/141/2015 (H5N1) LPAIV was isolated from a healthy wild mallard captured in 2015 and belonged to Eurasian, non-GsGD lineage (EPI_ISL_234408) [25]

c) A/common gull/Poland/MW241/2011 (H13N6) LPAIV was detected in a live common gull captured in 2011 (EPI_ISL_15429940).

All viruses were propagated in the allantoic cavity of embryonated 9–11-day-old specific pathogen free (SPF) chicken eggs (Valo Biomedia, Germany) and titrated according to standard procedure [26]. Allantoic fluid from infected eggs was diluted in phosphate buffered saline to obtain a final titer of 10^7^ median egg infectious doses (EID_50_) per mL. The challenge virus doses were checked by re-titration of the inoculum on embryonated SPF eggs.

### Birds

Herring gulls at 2–3 weeks of age were hand-caught in several breeding colonies located on the Baltic coast near the town of Łeba (Poland). Captured chicks were kept in an aviary until 4 weeks of age and then transported to the NVRI, where they were placed in the biosafety level 3 (BSL-3) animal facility, in rooms specifically adapted to the conditions required by gulls (i.e. special bedding, water tanks for bathing, boxes filled with cotton jute imitating nests, and wooden pallets allowing the birds to shelter). Access of the animals to feed and water was ad libitum.

Oropharyngeal and cloacal swabs were collected from each gull before inoculation. These samples were tested primarily for active AIV infection, but also other viral (parvovirus, rotavirus, astrovirus, adenovirus, coronavirus), bacterial (*Salmonell*a spp.) and parasitic (trematodes and nematodes) infections/infestations (methodology available upon request). Additionally, serum samples were also collected and checked for the presence of antibodies against AIV.

### Experimental design

Thirty-six herring gulls were divided into three experimental groups (A-C, 12 birds per group). All inoculated birds received the respective virus (LPAIV and/or HPAIV) at the dose of 10^6^ EID_50_ per bird according to the following scheme:

*Group A*: 8-week-old gulls inoculated with A/herring gull/Poland/MB082B/2016 (H5N8) HPAIV via intraocular and intranasal route (0.1 mL /bird);

*Group B*: 5–7-week-old gulls inoculated intraocularly, intranasally and *per os* with 1 mL/bird of A/mallard/Poland141/2015 (H5N1) LPAIV followed by inoculation with A/herring gull/Poland/MB082B/2016 (H5N8) HPAIV via intraocular and intranasal route (0.1 mL/bird) 2 weeks later;

*Group C*: 5–7-week-old gulls inoculated intraocularly, intranasally and *per os* with 1 mL/bird of A/common gull/Poland/MW241/2011 (H13N6) followed by inoculation with A/herring gull/Poland/MB082B/2016 (H5N8) HPAIV via intraocular and intranasal route (0.1 mL/bird) 2 weeks later.

Four gulls (group A and B) or three gulls (group C) were added to infected birds 24 h after inoculation with H5N8 HPAIV and they served as control of direct-contact virus transmission.

The gulls were observed daily for clinical signs and death. The body temperature was measured daily for a period of 14 days after HPAIV H5N8 infection. Oropharyngeal and cloacal swabs (Copan, Italy) were collected on day 2, 4, 7, 10 and 14 dpi (in the case of contact gulls: 1, 3, 6, 9 and 13 days postcontact—dpc) with HPAIV, immersed in viral transport medium and stored at −80 °C until further use. Blood was collected from gulls at 12 dpi for serologic testing (hemagglutination inhibition test and ELISA).

Additionally, two birds were sacrificed at 4 dpi and 14 dpi, necropsy was performed and selected organs were harvested for histopathological and immunohistochemical examinations (brain, trachea, lung, heart, liver, spleen, proventriculus, pancreas, duodenum, ileum and kidneys) as well as for viral RNA quantifications by real time RT-PCR (brain, lung, heart, liver, spleen, pancreas, duodenum and kidneys).

### Assessment of viral RNA quantity

The quantification of virus shed by oral/cloacal route of infected birds as well as its load in tissues was performed by quantitative real time RT-PCR (qrRT-PCR). Total RNA was extracted using Viral Mini Kit Plus (Syngen, Poland) following supplier’s instructions. The detection and quantification of virus load was conducted using QuantiTect Probe RT-PCR Kit (Qiagen, Germany) with primers and probe specific to the matrix (M) gene [27]. To generate a standard curve, ten-fold dilutions of a sample with a known number of the M gene copies were prepared and tested in parallel with the samples obtained from birds. The results were expressed as the number of gene copies per 0.2 mL of swab medium or organ homogenate.

### Histopathology and immunohistochemistry (IHC)

The brain, trachea, lung, heart, liver, spleen, proventriculus, pancreas, duodenum, ileum and kidney tissue samples collected during the necropsy were fixed in 10% neutral buffered formalin and processed routinely to paraffin blocks. Sections (4 µm) were stained with hematoxylin and eosin (HE) for histopathological examination. The same tissue sections were used for immunohistochemistry examinations. Before IHC staining slides were deparaffinized and subsequently subjected to a procedure aimed at exposing viral epitopes. Tissue section immunostaining was performed using 1:50 diluted anti-influenza A NP protein monoclonal antibody (HYB 340–05, Statens Serum Institute, Denmark) for 1 h at room temperature. After washing, the sections were treated with anti-mouse secondary antibody, horseradish peroxidase and DAB chromogen according to producer protocol (Dako Envision + , Dako, UK). Finally, tissue sections were counterstained with hematoxylin and examined microscopically. Recently proposed semiquantitative scoring system for lesions assessment was then applied [28].

### Serologic assays

At different time-points during the experiment (Table 2), sera from gulls were tested by blocking ELISA (AI Multi-Screen Ab Test, Idexx Europe B.V., Netherlands) and hemagglutination inhibition (HI) assay using 4 hemagglutinating units of the homologous antigens prepared from A/mallard/Poland/P141/2015 (H5N1), A/herring gull/Poland/MB082B/2016 (H5N8) and A/common gull/Poland/MW241/2011(H13N6), according to recommended protocol [29]. For HI test, sera exhibiting titers  ≥ 16 (≥4 log_2_) were considered positive.

### Statistical analysis

Survival analysis was performed using the Kaplan–Meier method based on data from experimental groups A, B and C. In addition, survival in all three groups was compared using the chi-square test and all groups were compared with each other using the Bonferroni correction and the following tests: Gehan the generalized Wilcoxon test, Cox-Mantel, log-rank, F Cox, and Peto & Peto version of the Wicoxon test. TIBCO Software Inc. (2017) Statistica (data analysis software system), version 13, was used for the analyzes. The data obtained from quantitative analysis of virus load in swab samples and body temperature were analyzed by nonparametric Kruskal–Wallis test. Results with *P* < 0.05 were considered as statistically significant.

## Results

### Clinical signs and mortality

Prior to experiment, all gulls were clinically healthy and none of the birds were positive for AIV by rRT-PCR or had detectable levels of antibodies against the AI virus. The panel of laboratory tests did not reveal the presence of common avian pathogens apart from deltacoronaviruses found in a few individuals, for which *Laridae* appear to be asymptomatic hosts [30]. Trematode and nematode eggs (unidentified species) were detected by parasitological examination. The body temperature before inoculation ranged from 40.6 to 41.5 °C.

In group A (infection with H5N8 HPAIV), the first clinical signs occurred within 24 h and lasted until the end of experiment. The following clinical manifestations were noted: increased body temperature, depression and unresponsiveness, absence of vocalization, recumbency, inappetence, opisthotonus, torticollis, head tremors, paralysis, conjunctivitis, dyspnea, nystagmus, diarrhea. The virus was highly lethal for gulls, as 11 infected birds died (or were humanely euthanized to terminate suffering) between 2 and 7 dpi and two contact gulls died at 3 and 7 dpc (Additional file 1). From day 7 pi onwards, a gradual improvement of health was observed in surviving gulls evidenced by increased appetite, vocalization, mobility and aggressive behavior towards handlers. In the second week of experiment, symptoms in the surviving birds were mostly restricted to conjunctivitis and bloody diarrhea. The body temperature raised in the first 3 days post infection reaching its peak at 3 dpi and then began to steadily fall. The Kruskal–Wallis test confirmed variation over time (*P* < 0.05) (Additional file 2). At necropsy, we found congestion of internal organs (mostly intestines and lungs) and hemorrhages in subcutaneous tissues from head, cerebral hemispheres, proventriculus, bursa of Fabricius, kidneys, liver and spleen, although not all birds showed obvious lesions.

**Figure 1:**
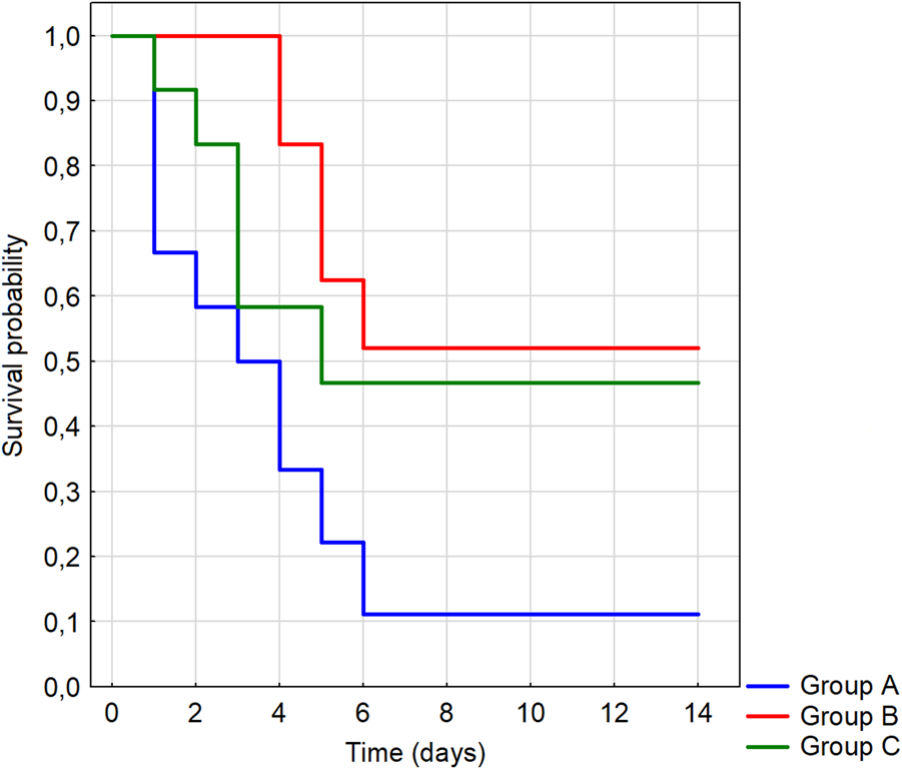
**Kaplan–Meier analysis of survival in three experimental groups of gulls infected with HPAIV H5N8 (group A), LPAIV H5N1 followed by HPAIV H5N8 (group B) and LPAIV H13N6 followed by HPAIV H5N8 (group C)**.

In group B (infection with H5N1 LPAIV followed by H5N8 HPAIV), clinical signs in gulls infected with LPAIV were not observed. Following infection with H5N8 HPAIV, the recorded clinical signs were present in all birds but appeared later than in group A (i.e. at 4 dpi) and included increased body temperature (variation over time was statistically significant, *P* < 0.0001), depression and absence of vocalization, uneven walk, inappetence, opisthotonus, shortness of breath. Five birds from directly inoculated group died between 5 and 7 dpi and 3 contact gulls succumbed between 7 and 10 dpc. All statistical tests used to analyze survival indicated statistically significant difference in survival between group A and B (*P* = 0.0051 to *P* = 0.0097) (Figure 1). From 8 dpi onwards, an improvement of health was observed: the birds were more difficult to handle, tried to fly away, readily used the water tanks, showed increased appetite. At necropsy, congested liver, pancreas, intestines (duodenum), spleen and sometimes proventriculus as well as petechiae in proventriculus and spleen were observed. In general, gross lesions were milder than those seen in gulls from group A.

In the experimental group C, two gulls were clearly depressed for 3 days from 5 days post infection with H13N6 LPAIV but no other explicit clinical symptoms were observed. The first clinical signs after HPAIV H5N8 infection were observed within 2 dpi and included loss of appetite and increased body temperature (variation over time was statistically significant, *P* < 0.0001). A total of 6 gulls died between days 2 and 6 pi with the highest number of dead birds on days 3 and 4 pi. As in groups A and B, the birds showed strong neurological and respiratory symptoms (shortness of breath) before death. None of the statistical tests showed significant differences in survival between groups A and C or B and C. Morbidity and mortality were not observed in contact gulls. From 10 dpi, a gradual improvement of health condition was observed in surviving gulls (four infected and three contact individuals). At necropsy, the major lesions were in general similar to those in group B with congestion of organs being the most prominent lesions. Mortality progress in all experimental groups is presented in Additional file 1.

### Virus quantity in swabs and selected tissues

**Figure 2:**
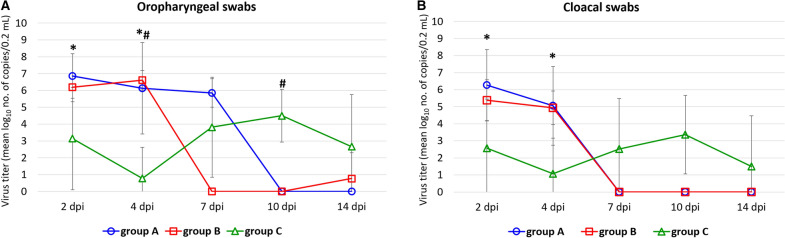
Mean virus shedding from respiratory and digestive tract of H5N8 HPAIV infected (A, B) gulls with preexisting homo- and heterologous immunity * and # denote significant differences between gulls from A and C groups and from B and C groups, respectively (P < 0.05).

The shedding of HPAIV H5N8 from the respiratory and digestive tract of gulls in groups A–C is shown in Figure 2. Due to small sample size of groups containing contact birds, statistical analysis was only performed for experimentally infected groups.

The data for experimental group A was limited due to high mortality and early termination of the experiments. When comparing the level of shedding between groups A-C, the statistically higher level of shedding, both from respiratory and intestinal tracts, was noted in the case of group A in comparison with group C at 2 and 4 dpi. Additionally, the gulls in group B shed higher amounts of virus from oral cavity than gulls from group C at 4 dpi, but at 10 dpi significantly higher virus quantities were confirmed in birds from group C in comparison with group B (*P* < 0.05). Transmission to contact gulls was observed in all groups with the onset of shedding observed at 4 dpi (group A and B) and 7 dpi (group C). The duration of shedding was up to 7 dpi in group A (experimental and contact gulls), up to days 4 and 14 pi in group B (experimental and contact gulls, respectively) and up to 14 day pi in group C (experimental and contact gulls). Three gulls in the group C showed a delayed onset of shedding.

The influenza virus was found in all or most of analyzed organs from H5N8 HPAIV infected gulls with none and preexisting homologous immunity (groups A and B) euthanized at 4 dpi which indicating a generalized infection (Table 1). Surprisingly, no virus was detected in the studied organs of gulls with preexisting heterologous immunity (group C) necropsied at this time point. The results of the virus content in the same tissues collected from gulls necropsied at the end of experiments were different. No viral RNA was found in organs of the gull from group A that survived until the end of experiment. Birds with the preexisting homologous immunity of group B had lower amounts of virus and only in some organs. Only one of the gulls had a systemic infection—the virus was detected in almost all organs (except the liver). In the remaining birds, the virus was detected most often in the duodenum and spleen (in 4 out of 5 examined birds). In other organs (brain, kidneys, lungs) its occurrence in other birds was rather occasional. In group C of birds with heterologous immunity, the HPAIV was detected in almost all organs of two gulls, only in duodenum and spleen of third gull but the fourth bird was free of virus presence.

**Table 1:** **Comparison of AIV titers in selected organs of gulls**

Group	Lung	Spleen	Brain	Heart	Kidney	Duodenum	Pancreas	Liver
4 days post-infection (virus titer* [no positive/no total])								
A	8.1 (2/2)	6.7 (2/2)	9.8 (2/2)	8.2 (2/2)	6.2 (2/2)	6.0 (2/2)	6.4 (2/2)	5.1 (2/2)
B	6.3 (2/2)	4.8 (2/2)	5.9 (2/2)	5.4 (2/2)	4.6 (2/2)	5.4 (2/2)	3.0 (1/2)	5.2 (2/2)
C	–(0/2)	–(0/2)	–(0/2)	–(0/2)	–(0/2)	–(0/2)	–(0/2)	–(0/2)
14 days post-infection								
A	–(0/1)	–(0/1)	–(0/1)	–(0/1)	–(0/1)	–(0/1)	–(0/1)	–(0/1)
B	2.0 (2/5)	3.3 (4/5)	1.6 (2/5)	0.9 (1/5)	1.5 (2/5)	3.2 (4/5)	0.8 (1/5)	–(0/5)
C	3.3 (3/4)	4.2 (3/4)	4.4 (2/4)	3.3 (2/4)	1.4 (1/4)	4.0 (2/4)	3.6 (2/4)	2.0 (2/4)

^*^AIV titers expressed as mean log_10_ of the number of viral copies per 0.2 mL of organ homogenate (at 4 dpi two gulls/at 14 dpi all survived were analyzed).

### Histopathology and immunohistochemistry

**Figure 3:**
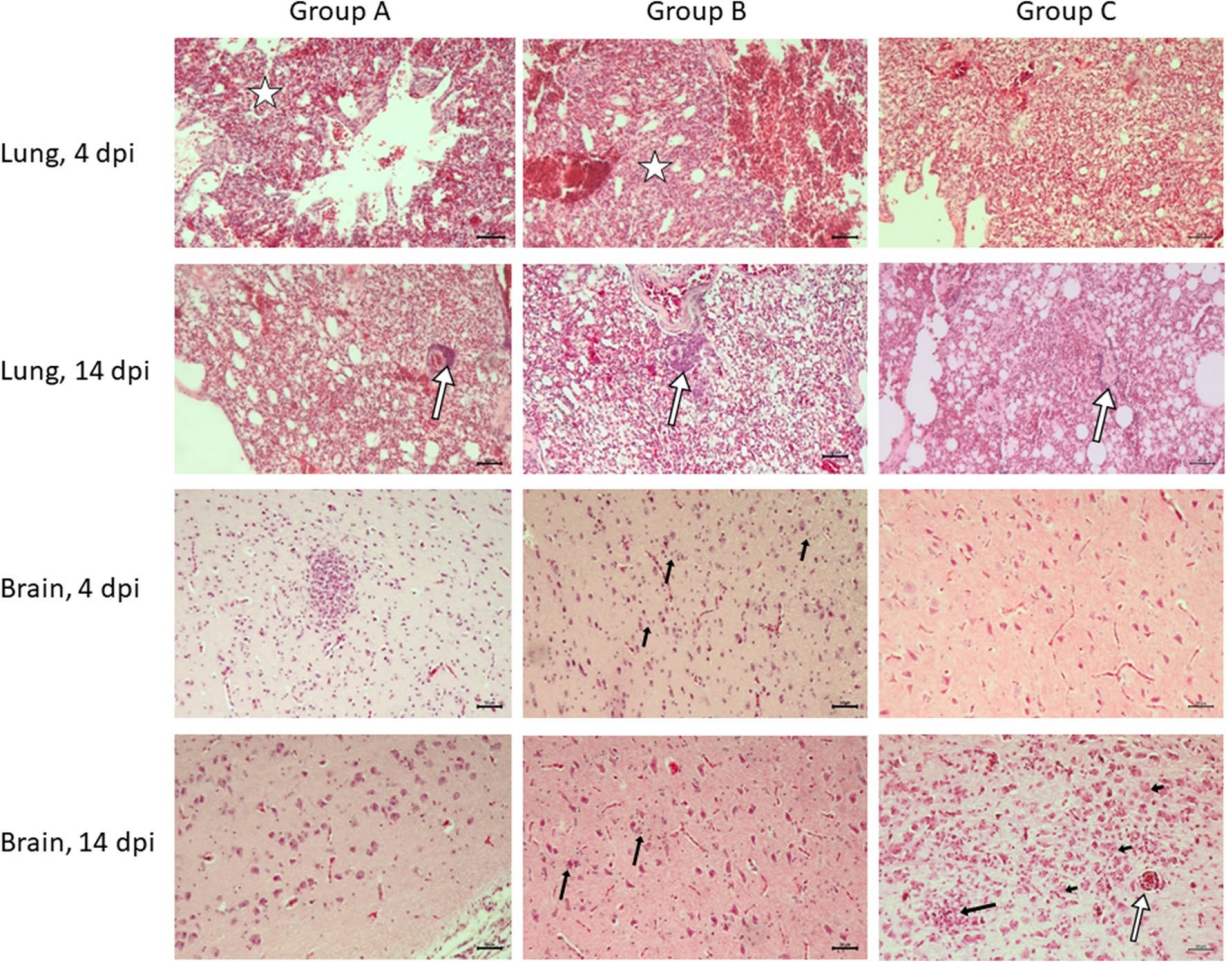
**Histopathologic results (hematoxylin and eosin staining) in lungs and brains collected from gulls representing groups A–C at 4 and 14 dpi**. Example photomicrographs, objective 10x. Lungs, 4dpi: congestion, oedema, fibrinous exudates and moderate necrosis within air capillaries (asterisk) in groups A and B, oedema in group C. Lung, 14 dpi: perivascular lymphoplasmatic infiltrations (white arrows) in groups A, B and C. Brain, 4 dpi, group A: glial nodule; group B: diffuse, moderate gliosis, activated microglia (black arrows), group C: mild gliosis; Brain, 14 dpi, group A: mild gliosis, group B moderate gliosis, aggregates of microglia around blood vessels and neurons; group C, severe encephalitis: there are multiple activated astrocytes visible surrounded by microglia (short arrows), glial nodules (black arrow), and perivascular lymphoplasmatic infiltrates (white arrow). Scale bar = 50 µm.

Generally, in gulls necropsied at 4 dpi, mainly necrotic changes in individual organs were observed. In turn, birds euthanized at 14 dpi usually showed lymphocytic infiltrates, while there were fewer necrotic lesions. As expected, microscopic lesions found in herring gulls of group A were the most severe and pronounced than in birds of other groups. In gulls infected with H5N8 HPAIV and necropsied at 4 dpi mild or moderate lesions were observed. In spleen, pancreas, jejunum, trachea and kidney these microscopic changes were only mild but moderate in proventriculus, liver, heart, brain and lungs. The lesion score increased to moderate or severe in gulls which were found dead. Nearly all gulls which died at 2 dpi (*n* = 4) or 3 dpi (*n* = 1) showed severe necrosis of lymphoid and reticuloendothelial cells in spleen, severe necrosis of parenchymal cells in lungs and necrotizing pancreatitis. The changes in the jejunum and the brain were assessed as moderate. The intensity of necrotizing encephalitis manifesting itself as randomly distributed focal areas of neuronal necrosis associated with gliosis and neuronophagia (Figure 3) was similar between the dead birds and the euthanized ones at 4 dpi. Interestingly, the only herring gull which survived up to 14 dpi showed only mild inflammatory lesions in most of the organs and no microscopic changes in brain, pancreas, trachea and jejunum.

No microscopic lesions or only these assessed as mild and rarely as moderate/severe necrotizing inflammation were observed in gulls from experiment B. No lesions were observed in proventriculus, pancreas and trachea and only mild ones in the rest of tested organs of both gulls euthanized at 4 dpi. The most impacted tissues were liver, heart and lungs where small necrotizing foci were found. Slightly more microscopic lesions were found in birds euthanized at 14 dpi. The most prominent changes presenting themselves as multifocal lymphohistiocytic infiltrates were observed in lungs and heart, whereas in brain areas diffuse gliosis was found (Figure 3). Moderate lymphoplasmatic infiltrates were present in livers.

In gulls of group C, similarly no lesions or mild ones were found in gulls euthanized at 4 dpi. However, more changes assessed as moderate or severe were observed in birds euthanized at 14 dpi. Severe microscopic lesions were found in brain (*n* = 3) and included neuronal degeneration, gliosis and perivascular lymphohistiocytic cuffing (Figure 3). Moderate segmental lymphohistiocytic infiltrates with focal necrosis were present in the lung (*n* = 2), heart (*n* = 2) and pancreas (*n* = 2). In other organs the lesions were mild.

**Figure 4:**
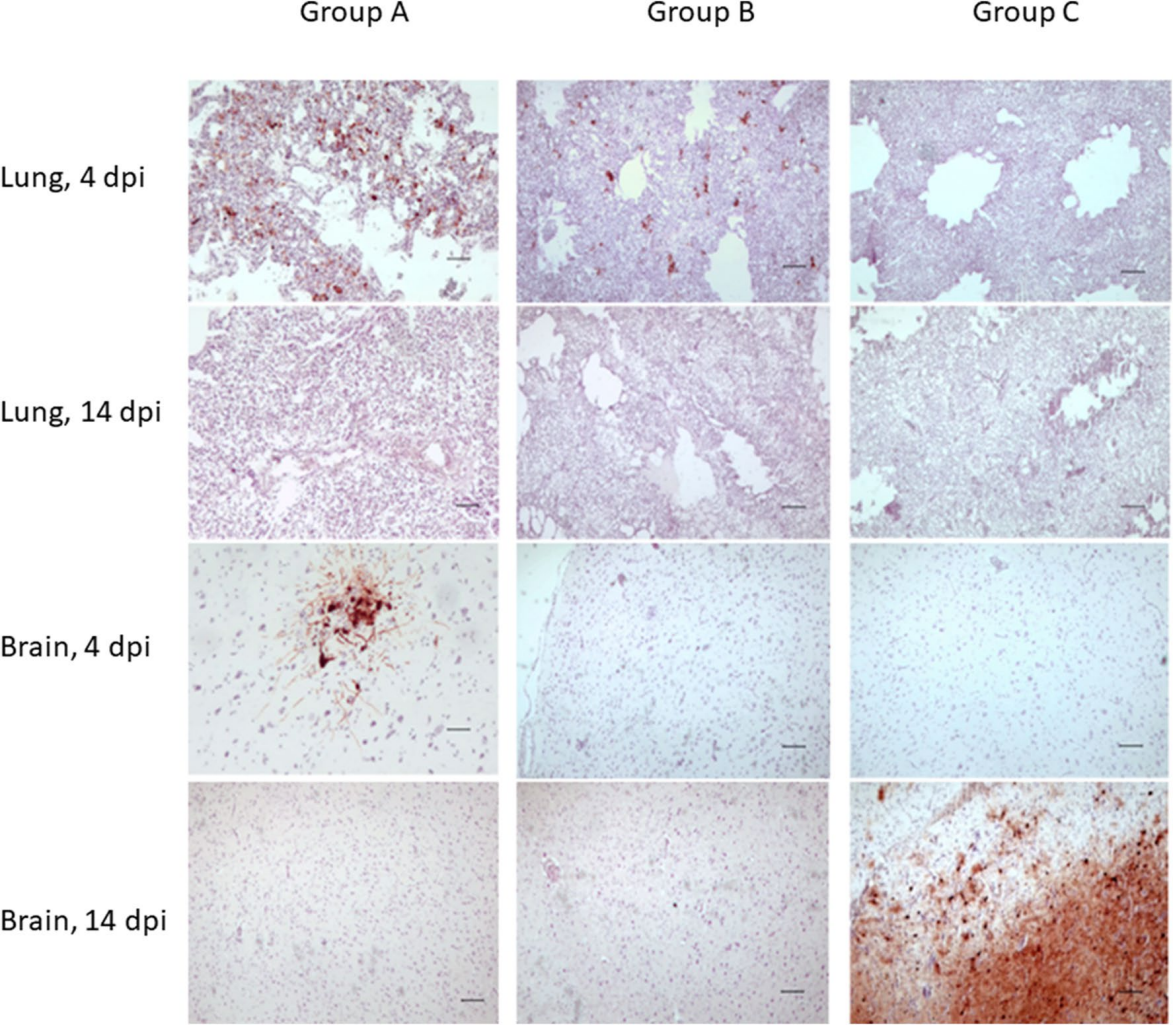
**Immunohistochemical staining of AIV nucleoprotein antigen in lungs and brains collected from gulls representing groups A–C at 4 and 14 dpi**. Example photomicrographs, objective 10x. Positive IHC reaction indicating the presence of AIV antigen is visible as dark-brown particles in the lungs from group A and B, 4 dpi, as well as in neuronal cells in brains from group A (4 dpi) and group C (14 dpi). Scale bar = 50 µm.

To follow the sites of virus replication, AIV antigen was immunohistochemically stained. In gulls inoculated only with the HPAIV (group A), high quantity of viral antigens were visible at 4 dpi in the areas with visible inflammatory-necrotic lesions i.e. in neural and glial cells in the brain, in respiratory capillaries (Figure 4), in myocardial fibers and in inflammatory infiltrates in the liver. In other tissues, despite severe histomorphological changes, immunolabelling was limited to singular macrophage like cells. There was no positive immunoreaction in any tissue from the only survivor at 14 dpi. The pattern of distribution of viral antigens observed in the gulls of group B necropsied on day 4 was similar in terms of the presence of NP antigens identified although its quantity was slightly lower than in group A. No presence of viral antigens was observed in tissues of group B birds sacrificed at 14 dpi, both the inoculated as well as contact gulls. On the other hand, no virus antigen was observed in the tissues of group C birds euthanized at 4 dpi, while high quantity of antigen was found in the brains of some birds necropsied at 14 dpi (Figure 4).

### Serology

At the onset of experiment, all gulls were negative by ELISA. Serological examination performed on sera collected from gulls infected with LPAIV H5N1 (group B) and H13N6 (group C) at the time of challenge with HPAIV H5N8 revealed 9/12 and 4/12 positive results (group B) as well as 3/13 and 0/12 positive results (group C) in HI and ELISA, respectively (Table 2). The HPAIV challenge induced strong humoral response in surviving birds and the mean geometric titer (log_2_) in seropositive gulls from group B and C were 8,8 and 8,7, respectively, reaching the highest values of 10 log_2_ (group B) and 9 log_2_ (group C). However, the only surviving birds in group A and one gull from group C did not develop antibodies that could be detected in either HI or ELISA.

**Table 2:** **Detection of antibodies to HA and NP proteins of AIV in sera herring gulls measured at different time-points of experiment**

Sampling time	Group A		Group B		Group C	
	HI^a^	ELISA	HI^a^	ELISA	HI^a^	ELISA
Day of challenge with LPAIV	–	–	Not tested	0/12	Not tested	0/12
Day of challenge with HPAIV	Not tested	0/12	9/12^b^ (GMT* 3.9 log_2_)	4/12	3/12^c^ (GMT 3.7 log_2_)	0/12
2 weeks post challenge with HPAIV	0/1	0/1	5/5^d^ (GMT 8.8 log_2_)	5/5	3/4^d^ (GMT 8.7 log_2_)	3/4

^a^values in brackets represent mean geometric titers (log_2_).

^b^tested with A/mallard/Poland/141/2015(H5N1) antigen.

^c^tested with A/common gull/Poland/MW241/2011(H13N6) antigen.

^d^tested with A/herring gull/Poland/MB082B/2016 (H5N8) antigen.

^*^geometric mean titer.

## Discussion

To our knowledge, this is the first report describing pathobiological characteristics of HPAIV infection in herring gulls with currently predominant clade 2.3.4.4b of H5 subtype (Gs/Gd lineage) and investigating the impact of homo- and heterosubtypic immunity on the outcome of infection with HPAIV.

In our study, we observed high virulence of HPAIV H5N8 clade 2.3.4.4b for naïve gulls as shown by early onset and severe clinical signs, including respiratory and neurological disorders as well as high mortality. Due to high mortality rate, the temporal analysis of viral shedding was strongly limited but revealed high amounts of the virus excreted both from oropharynx and cloaca. The analysis of virus presence in organs revealed its wide tissue distribution with the highest levels recorded in the brain, lung and heart. However, the relatively low intensity of antigen staining compared to high levels of viral RNA was found in the lungs. A similar inconsistency between antigen and virus quantities was observed in a previous study in Pekin ducks and can be explained by focal distribution of the virus in the organ or removal of blood containing high amounts of virus during the histological preparation of samples [31]. The obtained results, especially the predominant neurological disorders as well as inflammatory necrotizing lesions in the lungs, brain, heart, liver, digestive tract and pancreas (including generally higher intensity of necrotizing inflammation in the tissues from dead birds compared to the euthanized ones) are similar to those observed by other authors for herring gulls, laughing gulls and black-headed gulls infected with different clades of HPAIV H5N1 Gs/Gd [8–10]. The main conclusion of this subset of experiments is that herring gulls without pre-existing immunity to AIV are highly susceptible to infection with HPAIV H5N8 clade 2.3.4.4b, and they are expected to present with violent clinical outcome, high mortality, conspicuous nervous signs, systemic distribution of the virus in tissues, abundant viral shedding from both oral cavity and cloaca and transmission to susceptible individuals. It is of significance that one gull, despite showing overt clinical signs (depression, increased body temperature, bloody diarrhea) survived up to 14 days post infection and shed the virus at least up to 7 dpi but did not develop antibodies detectable by HI and ELISA tests.

On the other hand, prior infection of herring gulls with LPAIV H5N1 partially modulated the course of HPAIV H5N8 infection, as shown by statistically significant reduction of mortality, prolonged incubation period and rapid abrogation of shedding in convalescent birds. However, mortality and severe clinical signs, including neurological disorders, were still observed in some birds. Moreover, the amounts of H5N8 virus shed by infected gulls in the early phase of infection were high and the values, measured at the same time points, were not statistically different from those observed in group A. In order to rule out the possibility that positive rRT-PCR signals could have also come from residual LPAIV used for inoculation of gulls, we re-tested positive samples in another rRT_PCR using primers and probes targeting LPAIV subtypes used in the study (i.e. N1 in group B, H13 and N6 in group C) and obtained negative results. The virus was transmitted to naïve contact birds that also got sick and some of them died. The nature of lesions observed in histopathology in the brain, lungs, heart and liver at 4 dpi were similar to that in gulls from group A but had lower intensity. As the peer-reviewed literature is lacking reports describing the impact of homologous immunity on the outcome of HPAIV infection in gulls, we compared our results with similar published studies performed in wild ducks. For instance, wood ducks *Aix sponsa* pre-exposed to different strains of LPAIV H5 belonging to North American and Eurasian lineages showed, in general, reduced morbidity and mortality but no resistance to infection and replication of HPAIV H5N1 clade 2.2 [32]. On the other hand, captive mallards *Anas platyrhynchos* with previous homosubtypic infection with LPAIV H5 showed absence of disease and drastic reduction of shedding after challenge with HPAIV H5N1 (clade 1), that in turn induced severe disease and abundant shedding in control birds [33]. Based on our results, we conclude that pre-exposure of herring gulls to homologous subtype of LPAIV extends incubation period and increases survival rate after infection HPAIV H5 clade 2.3.4.4b but does not provide protection against disease and shedding, although might shorten its duration. The absence of virus shedding in convalescent gulls demonstrated in our study strongly limits their potential role as asymptomatic carriers of HPAIV after recovery. To what extent humoral and cell-mediated immunity directed against H5 subtype played a role in disease outcome’ modulation requires further elucidation.

In group C, infection with LPAIV H13N6, although asymptomatic in the majority of birds, induced mild and transient clinical signs (depression) in 3 individuals suggesting that gull-adapted LPAIV of H13 subtype is not completely benign and is able to cause disease in susceptible herring gulls. To our knowledge, there are no other reports on the course of infection with H13N6 subtype in herring gulls and scarce information on this subject in other gull species contrast with our findings [34]. After infection with HPAIV H5N8, although a conspicuous increase in survival was observed in comparison with group A, it was not confirmed by any of the statistical methods used. However, these results must be interpreted carefully since the small sample size (*n* = 12) increases the risk of type II error, i.e. when the null hypothesis is false but statistical tests fail to reject it. In comparison to group A, statistically significant lower level of virus shedding was noted at 2 and 4 dpi. That could be caused by the delayed onset of shedding in some birds, that were still rRT-PCR-positive by the end of experiment with the abundant presence of antigen detected by IHC at 14 dpi (at which time-point the results corresponded with the histopathological and IHC results observed in groups A and B at 4 dpi). The presence of asymptomatic shedders among convalescent birds raises epidemiological concerns and suggests that not only are herring gulls the victims of the virus but also can potentially play a role in virus perpetuation in the area and contribute to its spread to susceptible species. However, virus transmission observed in our experimental studies was only partially effective since contact gulls, despite some level of shedding, remained healthy and did not seroconvert. It is rather unlikely that the observed mitigation of clinical outcome of gulls infected with HPAIV H5N8 in group C was influenced by humoral immunity induced by exposure to H13N6 virus subtype. Firstly, very weak immune response was observed in gulls infected with H13N6 virus at the time of challenge. This phenomenon is hard to explain due to the paucity of scientific data on the onset and duration of humoral immunity in gulls. We hypothesize that the low number of seropositive gulls at the time of challenge in group C (and similarly, in group B) could have been caused by the short time span (2 weeks) between infection with LPAIV and HPAIV and that antibodies against LPAIV could take more time to develop up to the level detectable by diagnostic tests. This hypothesis needs further verification. Secondly, cross-reactivity of antibodies against H5 and H13 is hardly plausible; even among H13 subtype viruses the antigenic diversity is significant enough that some strains react poorly with antisera produced against other strains of this subtype [35]. It is possible that modulation of disease outcome in gulls pre-exposed to H13N6 LPAIV was induced by yet unidentified immunological mechanisms, such as pre-existing innate or cell-mediated immunity. Further studies are needed (qPCR for quantification of the level of IFN and other cytokines, flow cytometry) to better understand the underlying causes of this phenomenon.

There are a few caveats to the presented results. First, for practical reasons, the experiments were carried out in young gulls and some level of age-related resistance in adult birds is possible. However, field observations confirm that the overwhelming majority of positive herring gulls found in the past epizootics of HPAI in Europe were adult birds detected through passive surveillance that suggests that the virus was also lethal for older birds [11, 12]. The second caveat is relatively small number of birds used in the experiments that decreased the power of statistical tests and precluded the authors from drawing definitive conclusions for some of the analyses. Additionally, due to the very high frequency of reassortments between HPAIV H5Nx clade 2.3.4.4b and LPAIV, novel genotypes arising continuously can display slightly different biological properties for various species. For example, since late spring 2022, the recent reassortant HPAIV H5N1 has caused unusual mortality events in breeding colonies of terns and gulls in western Europe, including hundreds of herring gulls having been found dead in France [36], suggesting yet increased virulence in this species. Thus, enhanced surveillance combined with genomic characterization of detected viruses and, if necessary, in vivo studies, would facilitate the monitoring of changes in the pathogenic potential of currently circulating HPAIV.

To summarize, we conclude that herring gulls are highly susceptible to HPAIV H5N8 clade 2.3.4.4b and that pre-existing immunity to homo- and heterosubtypic LPAIV provides only some level of protection following exposure to HPAIV. However, prolonged duration of shedding observed in some birds does not rule out the possibility that they might contribute to the maintenance of the virus in a given area or even disseminate it over some distances. The histopathological and immunohistochemical results seem to confirm the pantropic nature of the HPAIV H5N8 clade 2.3.4.4b infection but with strong predilection of the virus to the neural, respiratory and myocardial tissues.

## Supplementary Information

**Additional file 1**. Number of gulls alive and spontaneously dead or euthanized during the experiments.

**Additional file 2**. Average body temperature of gulls in all experimental groups after H5N8 HPAIV infection.

## Data Availability

The datasets generated in the current study are included in the article, Additional files, or are available from the corresponding author on reasonable request.

## References

[1] Olsen B, Munster VJ, Wallensten A, Waldenstrom J, Osterhaus AD, Fouchier RA (2006). Global patterns of influenza a virus in wild birds. Science.

[2] Verhagen JH, Eriksson P, Leijten L, Blixt O, Olsen B, Waldenstrom J, Ellstrom P, Kuiken T (2021). Host range of influenza A virus H1 to H16 in Eurasian ducks based on tissue and receptor binding studies. J Virol.

[3] Verhagen JH, Fouchier RAM, Lewis N (2021). Highly pathogenic avian influenza viruses at the wild-domestic bird interface in Europe: future directions for research and surveillance. Viruses.

[4] Wille M, Barr IG (2022). Resurgence of avian influenza virus. Science.

[5] Bodewes R, Kuiken T (2018). Changing role of wild birds in the epidemiology of avian influenza A viruses. Adv Virus Res.

[6] Shriner SA, Root JJ (2020). A review of avian influenza A virus associations in synanthropic birds. Viruses.

[7] Arnal A, Vittecoq M, Pearce-Duvet J, Gauthier-Clerc M, Boulinier T, Jourdain E (2015). Laridae: a neglected reservoir that could play a major role in avian influenza virus epidemiological dynamics. Crit Rev Microbiol.

[8] Brown JD, Stallknecht DE, Swayne DE (2008). Experimental infections of herring gulls (*Larus argentatus*) with H5N1 highly pathogenic avian influenza viruses by intranasal inoculation of virus and ingestion of virus-infected chicken meat. Avian Pathol.

[9] Brown JD, Stallknecht DE, Beck JR, Suarez DL, Swayne DE (2006). Susceptibility of North American ducks and gulls to H5N1 highly pathogenic avian influenza viruses. Emerg Infect Dis.

[10] Ramis A, van Amerongen G, van de Bildt M, Leijten L, Vanderstichel R, Osterhaus A, Kuiken T (2014). Experimental infection of highly pathogenic avian influenza virus H5N1 in black-headed gulls (*Chroicocephalus ridibundus*). Vet Res.

[11] Adlhoch C, Fusaro A, Gonzales JL, Kuiken T, Marangon S, Niqueux E, Staubach C, Terregino C, Aznar I, Guajardo IM, Baldinelli F (2022). Avian influenza overview December 2021-March 2022. EFSA J.

[12] Brown I, Mulatti P, Smietanka K, Staubach C, Willeberg P, Adlhoch C, Candiani D, Fabris C, Zancanaro G, Morgado J, Verdonck F (2017). Avian influenza overview October 2016-August 2017. EFSA J.

[13] Lycett SJ, Duchatel F, Digard P (2019). A brief history of bird flu. Philos Trans R Soc Lond B Biol Sci.

[14] European Union Reference Laboratory for Avian Influenza, Padova, Italy (2022) Highly Pathogenic Avian Influenza (HPAI) in Europe: update. https://izsvenezie.com/documents/reference-laboratories/avian-influenza/europe-updates/HPAI/2021-1/wild-birds.pdf

[15] Sharshov K, Romanovskaya A, Uzhachenko R, Durymanov A, Zaykovskaya A, Kurskaya O, Ilinykh P, Silko N, Kulak M, Alekseev A, Zolotykh S, Shestopalov A, Drozdov I (2010). Genetic and biological characterization of avian influenza H5N1 viruses isolated from wild birds and poultry in Western Siberia. Arch Virol.

[16] Savic V, Labrovic A, Zelenika TA, Balenovic M, Separovic S, Jurinovic L (2010). Multiple introduction of Asian H5N1 avian influenza virus in Croatia by wild birds during 2005–2006 and isolation of the virus from apparently healthy black-headed gulls (*Larus ridibundus*). Vector Borne Zoonotic Dis.

[17] Del Hoyo J, Elliott A, Sargatal JE (1996). Handbook of the birds of the world Vol. 3. Hoatzin to Auks.

[18] Cramp S, Simmons KEL (1983). Handbook of the Birds of Europe, the Middle East and Africa. The birds of the western Palearctic. Vol. 3Waders to Gulls.

[19] Nogales M, Zonfrillo B, Monaghan P (1995). Diets of adult and chick herring gulls *Larus argentatus argenteus* on Ailsa Craig, south-west Scotland. Seabird.

[20] Huppop O, Wurm S (2000). Effects of winter fishery activities on resting numbers, food and body condition of large gulls *Larus argentatus* and *L. marinus* in the south-eastern North Sea. Mar Ecol Prog Ser.

[21] Garthe S, Wienck K, Cassens I (2000). Herring gull *Larus argentatus* winter diet at the western Baltic Sea coast: does ice cover make a difference?. Atl Seab.

[22] Goumas M, Boogert NJ, Kelley LA (2020). Urban herring gulls use human behavioural cues to locate food. Roy Soc Open Sci.

[23] Dalla Pria C, Cawkwell F, Newton S, Holloway P (2022). City living: nest-site selection preferences in urban herring gulls, *Larus argentatus*. Geographies.

[24] Swieton E, Smietanka K (2018). Phylogenetic and molecular analysis of highly pathogenic avian influenza H5N8 and H5N5 viruses detected in Poland in 2016–2017. Transbound Emerg Dis.

[25] Swieton E, Smietanka K (2017). Phylogenetic study of H5 low pathogenic avian influenza viruses detected in wild birds in Poland in 2010–2015. J Vet Res.

[26] Reed LJ, Muench H (1938). A simple method of estimating fiftly per cent endpoints. Am J Epidemiol.

[27] Spackman E, Senne DA, Myers TJ, Bulaga LL, Garber LP, Perdue ML, Lohman K, Daum LT, Suarez DL (2002). Development of a real-time reverse transcriptase PCR assay for type A influenza virus and the avian H5 and H7 hemagglutinin subtypes. J Clin Microbiol.

[28] Landmann M, Scheibner D, Graaf A, Gischke M, Koethe S, Fatola OI, Raddatz B, Mettenleiter TC, Beer M, Grund C, Harder T, Abdelwhab EM, Ulrich R (2021). A semiquantitative scoring system for histopathological and immunohistochemical assessment of lesions and tissue tropism in avian Influenza. Viruses.

[29] OIE (2021). Manual of diagnostic tests and vaccines for terrestrial animals chapter 3.3.4. “Avian influenza (infection with avian influenza viruses)”.

[30] Domanska-Blicharz K, Milek-Krupa J, Pikula A (2021). Diversity of coronaviruses in wild representatives of the *Aves* class in Poland. Viruses.

[31] Bingham J, Green DJ, Lowther S, Klippel J, Burggraaf S, Anderson DE, Wibawa H, Dong MH, Ngo TL, Pham PV, Middleton DJ, Daniels PW (2009). Infection studies with two highly pathogenic avian influenza strains (Vietnamese and Indonesian) in Pekin ducks (*Anas platyrhynchos*), with particular reference to clinical disease, tissue tropism and viral shedding. Avian Pathol.

[32] Costa TP, Brown JD, Howerth EW, Stallknecht DE, Swayne DE (2011). Homo- and heterosubtypic low pathogenic avian influenza exposure on H5N1 highly pathogenic avian influenza virus infection in wood ducks (*Aix sponsa*). PLoS ONE.

[33] Fereidouni SR, Starick E, Beer M, Wilking H, Kalthoff D, Grund C, Hauslaigner R, Breithaupt A, Lange E, Harder TC (2009). Highly pathogenic avian influenza virus infection of mallards with homo- and heterosubtypic immunity induced by low pathogenic avian influenza viruses. PLoS ONE.

[34] Verhagen JH, Hofle U, van Amerongen G, van de Bildt M, Majoor F, Fouchier RAM, Kuiken T (2015). Long-term effect of serial infections with H13 and H16 low-pathogenic avian influenza viruses in black-headed gulls. J Virol.

[35] Verhagen JH, Poen M, Stallknecht DE, van der Vliet S, Lexmond P, Sreevatsan S, Poulson RL, Fouchier RAM, Lebarbenchon C (2020). Phylogeography and antigenic diversity of low-pathogenic avian influenza H13 and H16 Viruses. J Virol.

[36] Adlhoch C, Fusaro A, Gonzales JL, Kuiken T, Marangon S, Niqueux E, Staubach C, Terregino C, Aznar I, Muñoz Guajardo I, Baldinelli F (2022). Avian influenza overview March–June 2022. EFSA J.

