# Supplementary material for: Pathogenicity of highly pathogenic avian influenza H5N8 subtype for herring gulls (Larus argentatus): impact of homo- and heterosubtypic immunity on the outcome of infection
Source: Vet Res. 2022 Dec 14;53:108. doi: 10.1186/s13567-022-01125-x (PMC9749649; doi:10.1186/s13567-022-01125-x)
Supplement: Supplementary file 1 — Additional file 1. Number of gulls alive and spontaneously dead or euthanized during the experiments. [file 13567_2022_1125_MOESM1_ESM.docx]

**Additional file 1**. **Number of gulls alive and spontaneously dead or euthanized during the course of experiments**

|  |  | **Number of birds** | | | | | | | | | | | | | |
| --- | --- | --- | --- | --- | --- | --- | --- | --- | --- | --- | --- | --- | --- | --- | --- |
|  | **Day post-inoculation/post-contact (dpi/dpc)** | **1/0** | **2/1** | **3/2** | **4/3** | **5/4** | **6/5** | **7/6** | **8/7** | **9/8** | **10/9** | **11/10** | **12/11** | **13/12** | **14/13** |
|  | H5N8 infected alive | 12 | 8 | 7 | 5 | 3 | 2 | 1 | 1 | 1 | 1 | 1 | 1 | 1 | 1 |
|  | H5N8 infected died/euthanized | 0 | 4 | 1 | 2 | 2 | 1 | 1 | 0 | 0 | 0 | 0 | 0 | 0 | 0 |
| Group A | H5N8 contact alive | 4 | 4 | 4 | 3 | 3 | 3 | 3 | 3 | 2 | 2 | 2 | 2 | 2 | 2 |
|  | H5N8 contact died/euthanized | 0 | 0 | 0 | 1 | 0 | 0 | 0 | 1 | 0 | 0 | 0 | 0 | 0 | 0 |
|  |  |  |  |  |  |  |  |  |  |  |  |  |  |  |  |
|  | H5N1+H5N8 infected alive | 12 | 12 | 12 | 10 | 8 | 6 | 5 | 5 | 5 | 5 | 5 | 5 | 5 | 5 |
|  | H5N1+H5N8 infected died/euthanized | 0 | 0 | 0 | 2 | 2 | 2 | 1 | 0 | 0 | 0 | 0 | 0 | 0 | 0 |
| Group B | H5N1+H5N8 contact alive | - | 4 | 4 | 4 | 4 | 4 | 3 | 3 | 2 | 1 | 1 | 1 | 1 | 1 |
|  | H5N1+H5N8 contact died/euthanized | - | 0 | 0 | 0 | 0 | 0 | 1 | 0 | 1 | 1 | 0 | 0 | 0 | 1 |
|  |  |  |  |  |  |  |  |  |  |  |  |  |  |  |  |
|  | H13N6+H5N8 infected alive | 12 | 11 | 8 | 5 | 5 | 4 | 4 | 4 | 4 | 4 | 4 | 4 | 4 | 4 |
|  | H13N6+H5N8 infected died/euthanized | 0 | 1 | 3 | 3 | 0 | 1 | 0 | 0 | 0 | 0 | 0 | 0 | 0 | 0 |
| Group C | H13N6+H5N8 contact alive | 3 | 3 | 3 | 3 | 3 | 3 | 3 | 3 | 3 | 3 | 3 | 3 | 3 | 3 |
|  | H13N6+H5N8 contact died/euthanized | 0 | 0 | 0 | 0 | 0 | 0 | 0 | 0 | 0 | 0 | 0 | 0 | 0 | 0 |
|  |  |  |  |  |  |  |  |  |  |  |  |  |  |  |  |
