# Supplementary figures and images for: Pathogenicity of highly pathogenic avian influenza H5N8 subtype for herring gulls (Larus argentatus): impact of homo- and heterosubtypic immunity on the outcome of infection
Source: Vet Res. 2022 Dec 14;53:108. doi: 10.1186/s13567-022-01125-x (PMC9749649; doi:10.1186/s13567-022-01125-x)

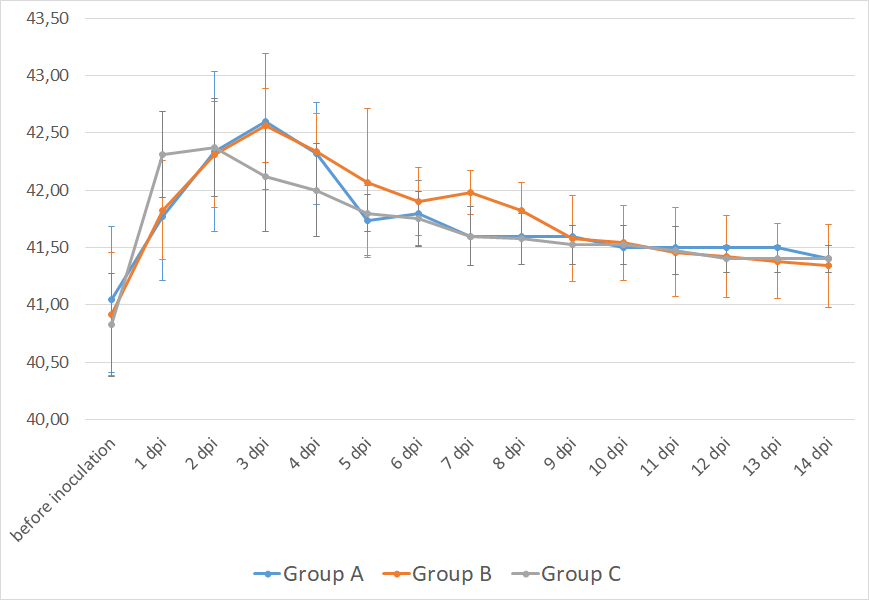

Supplement: Supplementary file 2 — Additional file 2. Average body temperature of gulls in all experimental groups after H5N8 HPAIV infection. [file 13567_2022_1125_MOESM2_ESM.tif]
